# Clinical Outcome Assessments Toolbox for Radiopharmaceuticals

**DOI:** 10.3389/fonc.2019.01028

**Published:** 2019-10-10

**Authors:** Charles A. Kunos, Jacek Capala, Adam P. Dicker, Benjamin Movsas, Susan Percy Ivy, Lori M. Minasian

**Affiliations:** ^1^Cancer Therapy Evaluation Program, National Cancer Institute, Bethesda, MD, United States; ^2^Radiation Research Program, National Cancer Institute, Bethesda, MD, United States; ^3^Sidney Kimmel Medical College, Thomas Jefferson University, Philadelphia, PA, United States; ^4^Henry Ford Hospital, Detroit, MI, United States; ^5^Division of Cancer Prevention, National Cancer Institute, Bethesda, MD, United States

**Keywords:** radiopharmaceutical, cancer, patient reported outcome (PRO), digital device usage, clinical outcome assessment, radiotherapy, radiotherapy adverse effects

## Abstract

For nearly 40 years, the U.S. National Cancer Institute (NCI) has funded health-related quality-of-life (HRQOL) and symptom management in oncology clinical trials as a method for including a cancer patient's experience during and after treatment. The NCI's planned scope for HRQOL, symptom and patient-reported outcomes management research is explained as it pertains to radiopharmaceutical clinical development. An effort already underway to support protocol authoring via an NCI Cancer Therapy Evaluation Program (CTEP) Centralized Protocol Writing Service (CPWS) is described as this service aids incorporation of HRQOL, symptom and patient-reported outcomes management research into sponsored protocols.

## Introduction

For nearly four decades, the National Cancer Institute (NCI) sponsored clinical trials have provided resources for research in health-related quality of life (HRQOL) and in symptom management for cancer patients (1). These resources have included infrastructure for cancer patient clinical trials that have symptoms as a primary end point, funding for investigator-initiated correlative studies involving HRQOL end points in late phase clinical trials, and grants studying the key issues and challenges facing investigators for implementing HRQOL and symptom management into its early phase clinical trials (1, 2).

Late phase clinical trials seek to improve cancer patient survival and more consideration has been given in these trials to the way in which cancer patients live during and after their treatments. A desire to meet HRQOL needs of cancer patients has incentivized NCI sponsored clinical trials to consider piloting the collection of HRQOL and patient-reported outcomes (PROs) by wearable digital technology like mobile phone applications or wristband sensors in parallel with its early phase clinical trials of radiopharmaceuticals. NCI sponsored clinical trials offer this strategic vision because radiopharmaceuticals have drug-like pharmacology in that these radioactive drugs have quantifiable pharmacokinetics, body weight-driven prescriptions, and predictable organ toxicities. Radiopharmaceuticals fit well into the programmatic mission of patient safety and symptom management for NCI sponsored clinical trials. Thus, integrating pilot HRQOL tools into early phase safety trials that are eventually intended to be used in late phase efficacy trials makes sense (3). Wearable digital technology in the form of mobile phone applications or wristband sensors captures in near-time the HRQOL and PRO data linked to acute toxicity, prompt and iterative symptom management, as well as reasons for treatment-related drug holiday or drug discontinuation (4).

The challenges and opportunities for integrating PRO and biometric endpoints into the roll-out of NCI sponsored radiopharmaceutical trials are discussed as the primary emphasis of this article. Opportunistic examples related to the Cancer Therapy Evaluation Program (CTEP) Centralized Protocol Writing Service (CPWS) and its incorporation of HRQOL, symptom and patient-reported outcomes management research into early-phase patient safety trials of radium-223 (Xofigo) or lutetium-177 dotatate (Lutathera) provide context for the discussion.

## Challenges and Opportunities

**Figure 1:**
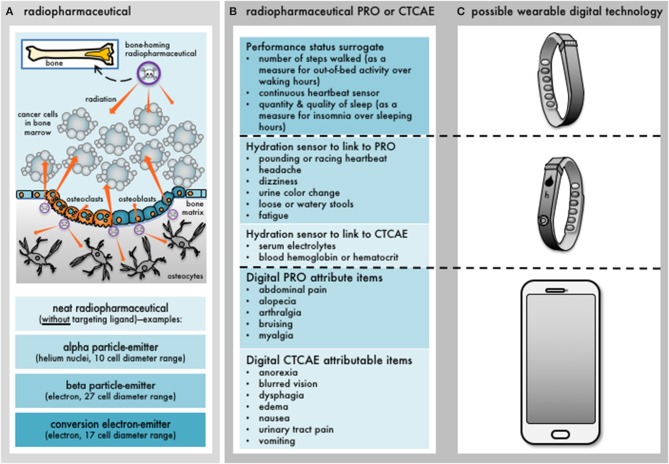
Development of digital patient-reported outcome measures for radiopharmaceuticals. Wearable digital technology is now commonplace among cancer patients. Patient-reported outcomes (PRO) or common terminology criteria for adverse events (CTCAE) items can be digitized for capture in the clinical development of radiopharmaceuticals. **(A)** Depicts an example of a bone-homing radiopharmaceutical intending to irradiate overlying cancer cells in the nearby bone marrow. It also shows that the radiopharmaceutical can irradiate osteocytes in a bone's mineralized matrix or bone marrow progenitor cells. Alpha particle-, beta particle-, or conversion electron-emitting radiopharmaceuticals would all have differential effects on normal cells depending on the range of irradiation. **(B)** Indicates what symptoms or clinical signs might manifest after multiple cycles of radiopharmaceutical administration. For example, PRO events like fatigue or CTCAE events like anemia that lessen overall performance status could become apparent. **(C)** Depicts some available commercial devices that quantifiably track activity or hydration status that might reflect some PRO or CTCAE items, or, digitally capture patient experiences in near real-time through applications or chatbots. Further research with wearable digital technology is needed.

From the time of cancer diagnosis to the end of life, cancer patients encounter a variety of functional and physical challenges (1). Undesirable outcomes from cancer or its treatment may range in scope from transient and reversible (for example, nausea or low white blood cell count), to cumulative (fatigue or abdominal pain), to subacute (3-month post-therapy cough from pneumonitis), or to late persistent and unremitting (dry mouth or vaginal dryness) (5). Pain, fatigue, and nausea are the most commonly encountered symptoms that occur along the trajectory of modern radiopharmaceutical treatment experience (6, 7). Cancer patients given radiopharmaceuticals may also have decreased appetite, vomiting, bruising easily, diarrhea, aching joints or muscles, or headache at various stages of their illness (6, 7). If not managed prospectively, a radiopharmaceutical-treated patient's physical, mental, or emotional well-being might be disrupted, thus impacting routine activities of daily living (Figure 1). Despite the long existence of these concepts (8), only now are “wearable” opportunities for symptom data collection becoming a reality (9). Wearable digital technology has evolved biometrics, or a capacity to observe, detect, and quantify, or in appropriate instances to intervene in, health parameters of the human body. Digital devices like wristband sensors now compute fitness and hydration level or out-of-bed activity and duration (Figure 1).

For many patient-reported symptoms, meaningful interventions have not been well-studied due to a scarcity of data on the incidence, prevalence, trajectory, and severity of symptoms (1). There is an imperfect knowledge of the physiologic mechanisms underlying symptoms altered by cancer treatments. NCI sponsored clinical trials offer a mechanism for scientifically and intellectually interesting radiopharmaceutical studies that incorporate HRQOL and PRO end points because they provide an opportunity safely and efficiently to study toxicity from the viewpoint of the patient in a near-time digital format. Currently, a number of projects to address HRQOL and PRO research gaps using digital technology are considered in NCI sponsored clinical trials. Indeed, digital technology might improve near real-time collection of HRQOL and PRO end points (10), but might also impact patient outcomes (11). Digital devices like a mobile phone application could capture near-time toxicity on patient-reported pain, fatigue, and nausea.

## Perspectives on Radiopharmaceutical Patient Reported Outcomes

From the outset, NCI sponsored clinical trials use an existing five-point scale Common Terminology Criteria for Adverse Events (CTCAE, version 5) toolbox for safety data and adverse event evaluation on radiopharmaceutical trials. While this method has limitations (Table 1), this approach builds upon prior notions that radiopharmaceutical-attributed toxicity falls into discrete toxicity categories that require medical instruments, technical training, or observable or subjective components (5, 12). For now, NCI investigators consider adverse events detected by instruments or those providers with technical training to follow CTCAE terminology and grading of severity. Adverse events that are subjective in nature with observable aspects (like radiation-induced diarrhea) or without observable qualities (like radiation-induced nausea) are amenable to patient reporting. Take for instance a trial participant's pretreatment grade 1 severity of frequent loose stools. On a trial evaluating the radiopharmaceutical radium-223 [a calcium mimetic eliminated via the relatively radiosensitive large intestine (13)], a participant's post-treatment severity of frequent loose stools might rise to grade 2, require antidiarrheal medication, and interfere with grocery shopping. CTCAE reports would capture the objective severity of loose stools requiring a physician-directed intervention in this case, but not necessarily the specific disruption of an instrumental activity of daily living. A PRO-CTCAE (v1.0) toolbox (14) incorporated into a radiopharmaceutical trial might improve the evaluation of this adverse event and provide the patient experience (Table 2). In this case scenario, capturing the patient's perspective on diarrheal frequency offers better qualified information on how an individual participant lives during and after their radiopharmaceutical treatment. For this reason, NCI investigators plan to list select toxicities like diarrhea as an adverse event of special interest when studying radium-223. As iterated elsewhere, an adverse event of special interest is a toxicity for which an expedited adverse event report must be filed to the NCI in its sponsored trials (5). PRO-CTCAE data have not been collected on radiopharmaceutical trials before, in part, because collection of such data is not common in early phase trials. Biometric data for trial endpoints (e.g., fasting glucose or specific changes in systolic and diastolic blood pressure parameters) are integrated in some NCI sponsored clinical trials.

**Table 1:** Complementary use of CTCAE and patient-reported outcome item formats for radiopharmaceuticals^*^.

	**CTCAE version 5 items**	**PRO-CTCAE version 1.0 items**
Primary utility	Report toxic effect of radiopharmaceutical	Report health status of patient
Best uses	Objective assessment (overt sign like hair loss)	Subjective assessment (obscure symptom like fatigue)
Best captures	Severity, for physician-directed intervention	Interference, for quality of life and treatment compliance
Validity	Not rigorously tested	Tested, with guidance for implementation (8)
Reliability	Not rigorously tested	Tested
Methods of data capture	Clinical interpretation, multilayered	Direct report from patient given radiopharmaceutical
Timing of data capture	Events occurs or at clinically-specified times	Evaluated at prespecified time points

* *Adapted from Bruner et al. (12). CTCAE, Common Terminology Criteria for Adverse Events*.

**Table 2:** Radiopharmaceutical patient-reported outcomes version of the CTCAE item formats^*^.

**Please think back over the past 7 days:**	**Example**
Severity (51 symptomatic AE terms): what was the severity of your _______ at its worse?	Abdominal pain (belly pain)
None/mild/moderate/severe/very severe	
Frequency (25 symptomatic AE terms): how often did you have _______?	Diarrhea (loose or watery stools)
Never/rarely/occasionally/frequently/almost constantly	
Interference (25 symptomatic AE terms): how much did _______ interfere with your usual activities?	Fatigue (lack of energy, tiredness)
Not at all/a little bit/somewhat/quite a bit/very much	
Presence (21 symptomatic AE terms): did you have any _______?	Bruising (black and blue marks)
No/yes	
Amount (2 symptomatic AE terms): did you have any _______?	Alopecia (hair loss)
Not at all/a little bit/somewhat/quite a bit/very much	

* *Adapted from Dueck et al. (14). AE, adverse events; CTCAE, Common Terminology Criteria for Adverse Events*.

Collection of biometric data or patient-reported outcomes in radiopharmaceutical trials is recommended. Investigators should consider employing the HRQOL instruments that measure, as optimally as possible, the relevant toxicity domains particularly relevant to the agent's mechanism of action (e.g., such as a radiopharmaceutical acting as a calcium mimetic and causing diarrhea), residence time (i.e., how long does a radiopharmaceutical “stick” to a target), and elimination from the body (like bowel or renal excretion inducing radiation-related enteritis or cystitis). A trial can incorporate the PRO instrument to provide information on specific symptoms or functional status, and any impact of the cancer and its treatment on HRQOL. Studies indicate that well-designed and well-conducted HRQOL research might guide future clinical trial design and morbidity end points by identifying certain patient conditions that variably confound HRQOL (14–16). For the best return on research investment, HRQOL research should detect HRQOL items both important to patients and likely to be impacted by the radiopharmaceutical intervention or the underlying cancer (1). As more trials find effective treatments, both patients and their physicians will want data on HRQOL and the influence radiopharmaceuticals will have on their physical health and functional performance.

Because of the ongoing discussions to incorporate HRQOL and symptom management in its randomized trials, NCI stakeholders have adapted CONSORT (consolidated standards of reporting trials) guidelines (17) for the reporting of radiopharmaceutical clinical trials that might incorporate such end points (Table 3). To date, there are no formal examples in which radiopharmaceutical trials have included HRQOL instruments. NCI stakeholders share their thoughts on this topic here as this sort of data in its trials should provide, to future patients and to their physicians, information regarding an expected course of radiopharmaceutical therapy alone or in combination. Such data should also define potential for recovery from radiopharmaceutical-related toxicity.

**Table 3:** Reporting radiopharmaceutical trials with patient-reported outcomes.

**Section**	**Item**	**CONSORT statement item**	**Radiopharmaceutical PRO item**
**TITLE AND ABSTRACT**			
	1a	Identify of radiopharmaceutical in trial title	Required for radiopharmaceutical trial
	1b	Structure a summary of design, methods, results, and conclusion	Indicate if PRO is primary or secondary aim
**INTRODUCTION**			
Background and objectives	2a	Provide radiopharmaceutical background and rationale	Provide rationale for PRO assessment
	2b	Specify hypotheses or clinical objectives	State specific PRO hypothesis and objective
**METHODS**			
Trial design	3a	Describe trial phase and design	Required for radiopharmaceutical trial
	3b	List methodological changes after trial commencement	
Participants	4a	List eligibility criteria for enrollees	List any PRO-related eligibility criteria
	4b	List locations of where data were collected	State PRO instrument, including how and
Interventions	5a	List radiopharmaceutical interventions	when they were assessed
	5b	List any non-radiopharmaceutical interventions	Cite PRO instrument validity and reliability
Outcomes	6a	Identify primary and any secondary outcome measures	List any PRO primary or secondary aim
	6b	List intervention changes after trial commencement	
Sample size	7a	State how sample size was calculates	Not required unless PRO is primary endpoint
	7b	Explain any interim analyses conducted or stopping rules executed	
**RANDOMIZATION**			
Sequence generation	8a	Specify methodology for random allocation	Option for radiopharmaceutical trial
	8b	Detail randomization type (such as blocking and block size)	List any PRO-related stratification factors
Allocation concealment	9a	Specify the mechanism for random allocation	State approach, if any
	9b	Specify any steps taken to conceal allocation until assignment	
Implementation	10a	List who generated the random allocation	State approach, if any
	10b	List who enrolled and assigned participants	
Blinding	11a	If done, state who was blinded to assigned interventions	State approach, if any
	11b	Describe any similarities of interventions	
Statistical methods	12a	Describe statistical methods to compare interventions	State approach for dealing with missing
	12b	List methods for any subgroup or adjusted analyses	PRO data in analyses
**RESULTS**			
Participant flow	13a	List numbers of participants assigned, treated, and analyzed	List numbers of participants at baseline
	13b	Identify numbers of participants excluded with reasons	and other timepoints for PRO data
Recruitment	14a	Define periods of trial recruitment and follow-up duration in the trial	
	14b	List when the trial ended, including reason(s)	
Baseline data	15a	Provide table of baseline demographics and clinical data	List any PRO-related eligibility criteria
	15b	List clinical indications for radiopharmaceutical administration	
Numbers analyzed	16a	List the number of participants (denominator) in analyses	Detail each PRO domain and time point
	16b	Describe if the analysis was by original assigned groups	Required for radiopharmaceutical trial
Outcomes estimation	17a	State effect size and precision (like 95% confidence interval)	
	17b	List absolute and relative effect for binary statistical outcomes	
Ancillary analyses	18a	Provide any subgroup ancillary analyses including PRO	Required for radiopharmaceutical PRO
	18b	Distinguish between prespecified from exploratory analyses	
Harms	19a	Report any harms or unintended toxicity effects in each group	Required for radiopharmaceutical PRO
	19b	Distinguish between prespecified from exploratory analyses	
**DISCUSSION**			
Limitations	20a	Discuss limitations, addressing potential bias or imprecision	Discuss radiopharmaceutical PRO-specific
	20b	Discuss any multiplicity of analyses	limitations
Generalizability	21a	Discuss generalizability of results considering prior evidence	Discuss radiopharmaceutical PRO-specific
	21b	Discuss external validity and applicability of trial findings	generalizability
Interpretation	22a	Interpret findings, balancing benefits and harms of intervention	Interpret radiopharmaceutical PRO in
	22b	Consider summary of other relevant evidence for context	relation to clinical outcome and survival
**OTHER INFORMATION**			
Registration and protocol	23	Provide number of trial registry, list if protocol can be accessed	Required for radiopharmaceutical trial
Funding	24	Indicate source of funding or support, identify role of funders	Required for radiopharmaceutical PRO

*Adapted from Calvert et al. (17). PRO, patient-reported outcome.

Digital therapeutics provide another opportunity for advancements (18). These interventions are often pushed onward to the health consumer by high-quality software programs that integrate protocol-defined management steps to prevent, manage, or treat a medical disorder or a disease like cancer (18). Independently or together with medications, devices, or other therapies, digital therapeutics raise the “quality” level of patient care for enhanced health outcomes (18). At present, digital therapeutic devices are expected to incorporate best health industry practices relating to design, clinical testing, usability, and personal data security (18). Regulatory bodies now recognize digital therapeutics as a means to support drug product claims for risk, efficacy, and intended clinical indication (18). Digital therapeutics allow patients, healthcare providers, and payers to have smart and handy tools to address health conditions through high-quality, safe, and effective data-driven interventions (18). One mechanism that the NCI might use to write-in digital therapeutics in its trials is a centralized protocol writing service.

## Perspectives on a CTEP Centralized Protocol Writing Service

**Figure 2:**
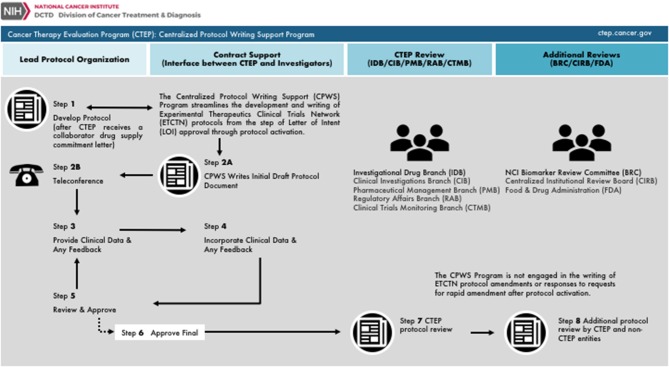
NCI Cancer Therapy Evaluation Program (CTEP) centralized protocol writing service. Charted is the workflow for the U.S. National Cancer Institute's CTEP Centralized Protocol Writing Service (CPWS) Program. From left to right, the chart is organized by the main protocol authoring entity involved in document writing inclusive of the Lead Protocol Organization, CTEP contract support (or CPWS), CTEP branches, or additional reviewers or other Federal agencies. Steps 1 and 2 initially activate CPWS protocol authoring. Steps 3 through 5 represent iterative and interactive feedback loops between CPWS and the principal investigator charged with protocol authoring. Step 6 represents a joint principal investigator and CPWS approval of the draft protocol. Step 7 and Step 8 involve scientific and logistical CTEP and non-CTEP reviews.

NCI CTEP launched a Centralized Protocol Writing Service (CPWS) to aid its Experimental Therapeutics Clinical Trials Network (ETCTN) investigators for streamlined development of clinical trial protocols (Figure 2). The CPWS offers this service for the initial clinical trial document development to support rapid protocol activation; it does not provide service for post-activation protocol amendments. NCI CTEP considers the principal investigator as the accountable leader of a clinical team, meaning they are the individual who interacts with the CPWS and who conducts the clinical investigation. NCI CTEP presumes of the principal investigator the role for protocol document oversight, the responsibility for delegation of written tasks, and the provision of responses to feedback from NCI CTEP, CPWS, or other regulatory agencies. After a CPWS kick-off teleconference, there are iterative and interactive feedback loops that are intended to incorporate scientific, clinical, procedural, logistical, or regulatory items in a clinical trial protocol document (Figure 2). Once reviewed and approved by the principal investigator and the CPWS team, NCI CTEP provides final review and obtains any need additional reviews prior to actual protocol activation. As of March 2019, two radiopharmaceutical clinical trial protocols for radium-223 (Xofigo) were written by ETCTN principal investigators and the CPWS. Protocol authoring by the CPWS took an average 33 days, compared to a 60-day target. The CPWS will be engaged in writing lutetium-177 dotatate (Lutathera) radiopharmaceutical clinical trial protocols in the near-term future.

## Conclusion

NCI sponsored clinical trials have supported the growth and execution of HRQOL and symptom management studies into clinical trials through a variety of pilot opportunities as part of protocol development. NCI investigators and stakeholders appreciate that early phase clinical trials evaluate the safety, and perhaps efficacy, of cancer treatment interventions among a diverse spectrum of cancer disease stages. In some instances, like the clinical development of radiopharmaceuticals, it makes sense to incorporate HRQOL and/or PRO tools in the early evaluation of agent safety when there is an anticipated impact collectively on patients, their caregivers, and their family members. This type of research can provide valuable data to patients, investigators, and regulators in early phases of clinical development before launching late phase clinical trials. The new NCI CTEP CPWS provides early phase trial investigators a means for iterative and interactive protocol writing, which may include HRQOL or PRO assessments in NCI sponsored clinical trials.

## Ethics Statement

The research presented in this article involved the collection or study of existing data, documents, and records that were publicly available. The research is regarded exempt from Institutional Review Board oversight.

## Author Contributions

CK, JC, AD, BM, LM, and SI contributed to the collection and review of any perspective data, analysis, and authentication, and the writing and approval of this manuscript. The views expressed are those of the authors and not those of the U.S. Federal government. Links or discussion of specific radiopharmaceutical drug products do not constitute endorsement.

### Conflict of Interest

The authors declare that the research was conducted in the absence of any commercial or financial relationships that could be construed as a potential conflict of interest.
